# Serum PAI-1/BDNF Ratio Is Increased in Alzheimer’s Disease and Correlates with Disease Severity

**DOI:** 10.1021/acsomega.3c04076

**Published:** 2023-09-19

**Authors:** Francesco Angelucci, Katerina Veverova, Alžbeta Katonová, Martin Vyhnalek, Jakub Hort

**Affiliations:** † Memory Clinic, Department of Neurology, Second Faculty of Medicine, Charles University and Motol University Hospital, Prague 150 06, Czech Republic; ‡ International Clinical Research Centre, St. Anne’s University Hospital, Brno 602 00,Czech Republic

## Abstract

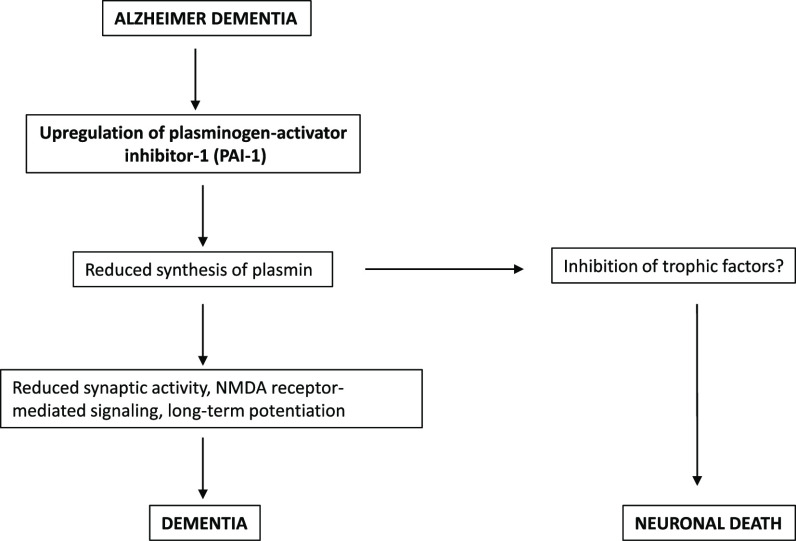

We previously demonstrated that serum levels of plasminogen activator inhibitor-1 (PAI-1), which inhibits both the tissue plasminogen activator (tPA) and plasmin activity, are increased in patients with Alzheimer’s disease. tPA/plasmin not only prevents the accumulation of β-amyloid in the brain but also is involved in the synthesis of the brain-derived neurotrophic factor (BDNF), a neurotrophin whose levels are reduced in Alzheimer. In the present study, we compared BDNF serum levels in Alzheimer patients with dementia to those in Alzheimer patients with amnestic mild cognitive impairment and to cognitively healthy controls. Moreover, we examined whether the PAI-1/BDNF ratio correlates with disease severity, as measured by Mini-Mental State Examination. Our results showed that BDNF serum levels are lower (13.7% less) and PAI-1 levels are higher in Alzheimer patients with dementia than in Alzheimer patients with amnestic mild cognitive impairment patients (23% more) or controls (36% more). Furthermore, the PAI-1/BDNF ratio was significantly increased in Alzheimer patients as compared to amnestic mild cognitive impairment (36.4% more) and controls (40% more). Lastly, the PAI-1/BDNF ratio negatively correlated with the Mini-Mental score. Our results suggest that increased PAI-1 levels in Alzheimer, by impairing the production of the BDNF, are implicated in disease progression. They also indicate that the PAI-1/BDNF ratio could be used as a marker of Alzheimer. In support of this hypothesis, a strong negative correlation between the PAI-1/BDNF ratio and the Mini-Mental score was observed.

## Introduction

Alzheimer’s disease (AD) is a progressive neurodegenerative disorder that affects millions of people worldwide.^1^ The hallmarks of Alzheimer are the presence of β-amyloid plaques and neurofibrillary tangles in the brain, which lead to the loss of neurons and synaptic connections.^2^ As a result, patients with Alzheimer experience cognitive decline and memory loss.^1^ Despite years of research, there is still no cure for Alzheimer’s disease, and treatments are only partially effective.

The brain-derived neurotrophic factor (BDNF) plays a key role in adult neurogenesis, as well as in plasticity and survival of neurons in the central nervous system (CNS).^3,4^ The BDNF is synthesized and secreted by various cell types, including neurons, astrocytes, and microglia.^4^ Several studies have reported a decrease in BDNF levels in the hippocampus, a brain region with a pivotal role in memory formation, in patients with Alzheimer.^5-7^ This decrease is a consequence of the accumulation of β-amyloid in neurons, which can impair BDNF signaling.^8^ However, regulation of BDNF expression and secretion is a complex process that involves several signaling pathways and molecular mechanisms. One of the mechanisms that regulates BDNF synthesis and secretion in the CNS is the plasminogen/plasmin system.^9-11^

Plasmin is a serine protease of the fibrinolytic system, which is responsible for the breakdown of blood clots.^12^ Plasmin is not only involved in clot degradation but also plays a role in various physiological and pathological processes in the CNS.^13,14^ Regarding cognitive functions, it has been observed that reduced fibrinolytic activity can increase the risk of mild cognitive impairment through mechanisms independent of β-amyloid.^15,16^ During transient ischemic attack, the disruption in blood supply in the brain causes a lack of oxygen, which may be responsible for development of transient^17^ or permanent^18^ mild cognitive impairment.

In addition to fibrinolytic activity, in the brain, plasmin and its tissue plasminogen activator (tPA) can cleave neurotrophin precursors to their mature form.^19^ It is known that neurotrophin precursors are not inactive. While proBDNF accelerates deposition of β-amyloid, favors long-term depression, and induces apoptosis,^20^ the BDNF inhibits the deposition of β-amyloid, favors long-term potentiation, and has an antiapoptotic effect.^21^ Thus, the role of tPA/plasmin in regulating BDNF synthesis and secretion is crucial for various physiological processes in the CNS, including synaptic plasticity, learning, and memory. For instance, the activation of tPA/plasmin, by inducing the conversion of proBDNF to the BDNF, can promote the formation and maintenance of synaptic connections in the hippocampus.^22^ In addition, tPA/plasmin can also regulate the expression of the BDNF by activating the mitogen-activated protein kinase (MAPK) signaling pathway, which is involved in the transcriptional regulation of the BDNF.^23^

In recent years, there has been growing interest in the role of plasmin in Alzheimer. Several studies have reported that plasmin activity is decreased in Alzheimer brains due to the inhibition of the enzymes that convert plasminogen to plasmin.^11,24,25^ This inhibition is believed to be mediated by the plasminogen activator inhibitor-1 (PAI-1), which is upregulated in Alzheimer and can impair the activity of the PA system.^26,27^ Accordingly, increased levels of PAI-1 in the brains and cerebrospinal fluid of patients with Alzheimer have been reported.^28-30^

In this study, we compared the serum levels of PAI-1 and BDNF in patients with Alzheimer’s dementia to those of patients with amnestic mild cognitive impairment and to controls. We also investigated whether the molar ratio between these two proteins is altered in these groups and whether this ratio correlates with the severity of cognitive impairment measured by the Mini-Mental score. We found that BDNF serum levels are lower (13.7% less) and PAI-1 levels are higher (23% more) in Alzheimer patients with dementia than in Alzheimer patients with amnestic mild cognitive impairment patients. Furthermore, the PAI-1/BDNF ratio was significantly increased in Alzheimer patients as compared to amnestic mild cognitive impairment (36.4% more) and controls (40% more). Lastly, the PAI-1/BDNF ratio negatively correlated with the Mini-Mental score.

## Methods

### Participants

Ninety participants from the database of the Czech Brain Aging Study^31^ were recruited between January 2018 and June 2021. Of them, 40 were diagnosed with dementia with clear evidence of Alzheimer’s disease in the pathophysiological process, 40 were diagnosed with amnestic mild cognitive impairment with high or intermediate likelihood of Alzheimer etiology,^32,33^ and 10 were cognitively healthy participants. At admission, all subjects were evaluated by standard neurological, neuropsychological, and biochemical analyses. Informed consent was signed by all subjects included in the study, and the protocol was approved by the ethics committee of Motol University Hospital in Prague.

### Exclusion Criteria

The following exclusion criteria were adopted: (1) past diagnosis of other neurological or psychiatric disorders potentially affecting cognitive functions (i.e., Parkinson’s disease, ischemia or stroke, alcohol addiction, and brain cancer), (2) hearing problems, (3) depressive symptoms (≥6 points on the 15-item Geriatric Depression Scale),^34^ and (4) vascular impairment at brain MRI (Fazekas scale more than 2).^35^

### Blood Sampling

Serum was obtained from venous blood after centrifugation at 2000*g* for 20 min. After centrifugation, serum was collected and stored at −80 °C.

### PAI-1 and BDNF Determination

Commercial ELISA kits from R and D Systems (Minneapolis, MN, USA) were used to determine PAI-1 (catalog number DY1786) and BDNF (catalog number DY248) serum levels. All measurements were executed in duplicate, and PAI-1 and BDNF levels were expressed as ng/mL.

### PAI-1/BDNF Ratio Determination

The PAI-1/BDNF molar ratio was calculated by using PAI-1 and BDNF serum levels with the formula PAI-1 (ng/mL):BDNF (ng/mL) = PAI-1/BDNF ratio.^26^

### Statistical Analysis

Univariate analyses of variance (ANOVA) were used to evaluate differences in PAI-1 and BDNF serum levels among the experimental groups. Post hoc tests were performed by Fisher-protected least significant difference tests. A chi-squared test was used for analysis of categorical data. Correlations between biochemical and clinical data were performed by Pearson correlation. A *p*-value of <0.05 was considered statistically significant.

## Results

### Demographic Characteristics

Demographic characteristics are shown in Table 1. There was no difference in sex distribution among the groups (chi-square *p*-value = 0.3). There was a significant group effect for age, indicating that the control group was younger than the amnestic mild cognitive impairment (*p* < 0.01) and Alzheimer (*p* < 0.01) groups. There was no difference in age between amnestic mild cognitive impairment and Alzheimer groups (*p* = 0.7).

**Table 1:** Clinical and Demographic Characteristics of Alzheimer’s Dementia Patients, Patients with Amnestic Mild Cognitive Impairment, and Healthy Subjects^a^

parameter	AD patients (*n* = 40)	aMCI patients (*n* = 40)	controls (*n* = 10)
age (years ± SD)	71 ± 9	70 ± 7	61 ± 12
male/female ratio (%)	12/28 (30%)	17/23 (42%)	2/8 (20%)
years of education	13.5 ± 2.9	14.8 ± 3.1	17 ± 1.65
Mini-Mental score	19 ± 4	25 ± 3	29.9 ± 0
PAI-1 (ng/mL)	6.1 ± 0.9	4.7 ± 1.5	3.9 ± 0.8
BDNF (ng/mL)	15.7 ± 3.9	18.2 ± 3.0	15.8 ± 3.0
PAI-1/BDNF ratio	0.41 ± 0.12	0.26 ± 0.08	0.24 ± 0.04

a SD: standard deviation; AD = Alzheimer’s disease; aMCI = amnestic mild cognitive impairment.

The Alzheimer and amnestic mild cognitive impairment groups had significantly lower years of education as compared to the control group (*p* < 0.001), while there was no difference in education between amnestic mild cognitive impairment and Alzheimer (*p* = 0.194). The Mini-Mental score was significantly lower in the Alzheimer (36.4% less) and amnestic mild cognitive impairment (16.4% less) groups as compared to controls (*p* < 0.001 for both comparisons). The Alzheimer dementia group also had a lower Mini-Mental score compared to the amnestic mild cognitive impairment group (24% less) (*p* < 0.001).

### Serum Levels of PAI-1 and BDNF in Alzheimer’s Dementia, in Patients with Amnestic Mild Cognitive Impairment, and in Controls

**Figure 1:**
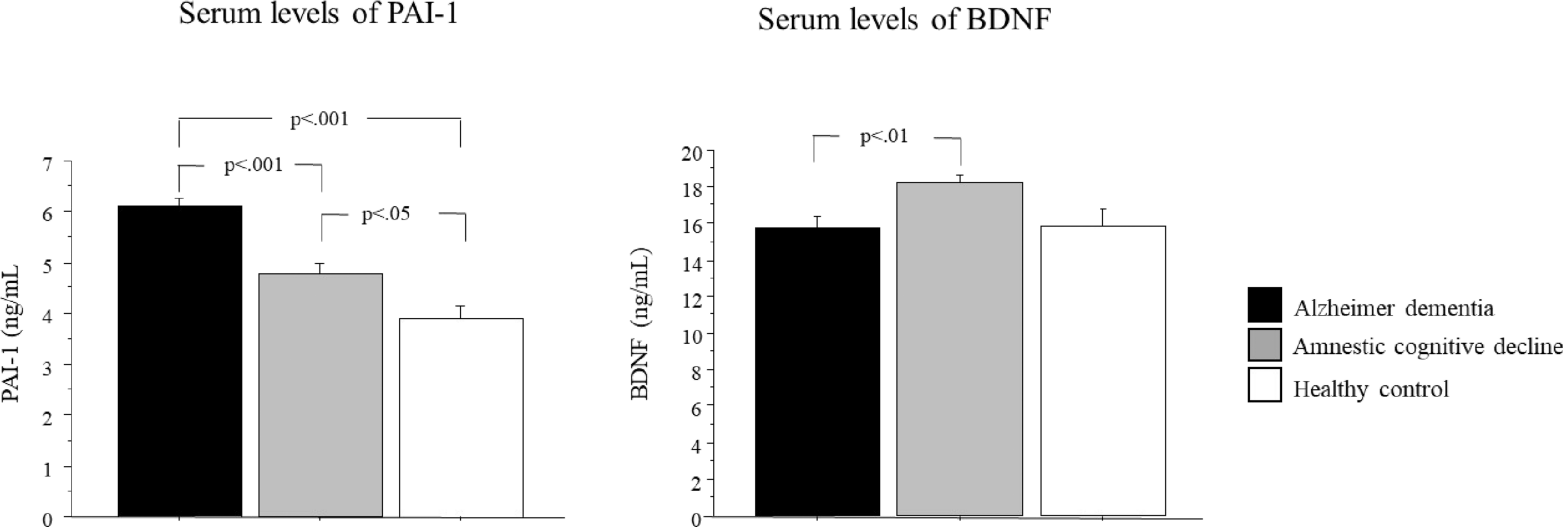
PAI-1 and BDNF serum levels in Alzheimer’s dementia, in patients with amnestic mild cognitive impairment, and in controls. Data are expressed as the mean ± standard error of the mean. Values are expressed in ng/mL (PAI-1 and BDNF).

Serum levels of PAI-1 and BDNF in Alzheimer’s dementia, in patients with amnestic mild cognitive impairment, and in controls are shown in Figure 1. There was a significant group effect in PAI-1 levels (*p* < 0.001). The post hoc analysis showed that PAI-1 levels were significantly higher in the Alzheimer group (23% more) as compared to amnestic mild cognitive impairment (*p* < 0.001; 95% CI: −2.02, −0.71) and controls (36% more) (*p* < 0.001; 95% CI: −3.27, −1.19). Moreover, the amnestic mild cognitive impairment group had significantly higher PAI-1 levels (17% more) as compared to the control group (*p* < 0.05; 95% CI: −1.90, 0.172) (Figure 1).

The results also showed a significant group effect in BDNF levels (*p* < 0.01). The post hoc analysis showed that BDNF levels were significantly higher in the amnestic mild cognitive impairment group as compared to the Alzheimer group (13.7% more) (*p* < 0.01; 95% CI: 0.66, 4.34) (Figure 1).

### PAI-1/BDNF Ratio in Alzheimer’s Dementia, in Patients with Amnestic Mild Cognitive Impairment, and in Controls

**Figure 2:**
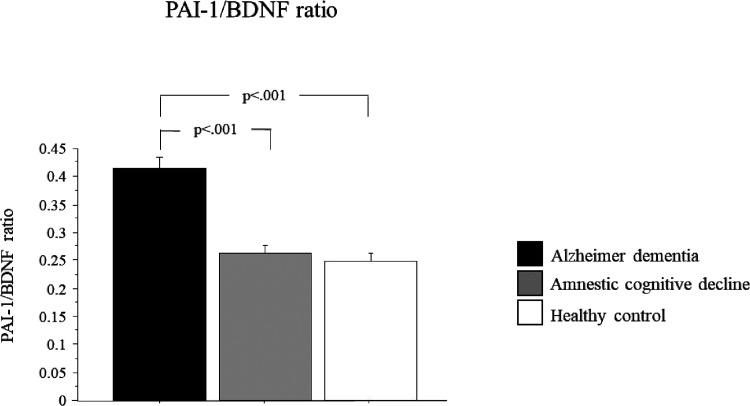
PAI-1/BDNF ratio in Alzheimer’s dementia, in patients with amnestic mild cognitive impairment, and in controls. Data are expressed as the mean ± standard error of the mean.

The ratio between the PAI-1/BDNF serum levels is shown in Figure 2. There was a significant group effect (*p* < 0.0001). Post hoc analysis showed that the PAI-1/BDNF ratio was significantly higher in the Alzheimer group as compared to amnestic mild cognitive impairment (36.4% more) (*p* < 0.0001; 95% CI: −0.2, 0.097) and controls (40% more) (*p* < 0.0001; 95% CI: −0.25, −0.082) (Figure 2).

### Correlations between the PAI-1/BDNF Ratio and Disease Severity

**Figure 3:**
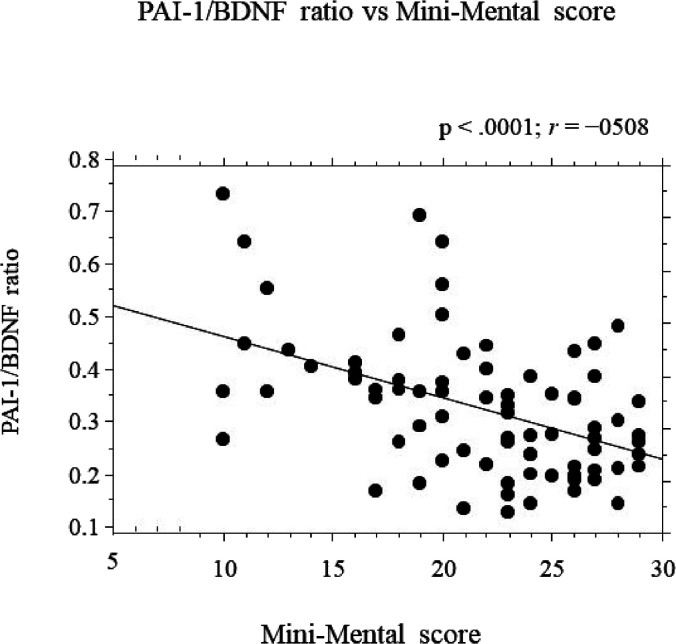
Correlations between the PAI-1/BDNF ratio and the Mini-Mental score. *r* is the Pearson correlation coefficient.

Correlation analysis of the PAI-1/BDNF ratio versus e Mini-Mental score is shown in Figure 3. We observed a strong negative correlation between the PAI-1/BDNF ratio and the Mini-Mental score (*r* = −0.508, *p* < 0.0001). That is, PAI-1/BDNF serum levels were negatively associated with Mini-Mental scores.

## Discussion

The mechanisms by which elevated levels of PAI-1 have been implicated in the pathogenesis of Alzheimer involve the accumulation of β-amyloid due to reduced tPA/plasmin production and reduced neurogenesis and loss of synaptic connections in the brain due to reduced BDNF signaling. In this study, we analyzed PAI-1 and BDNF serum levels and the PAI-1/BDNF ratio in patients with Alzheimer’s dementia, in individuals with amnestic mild cognitive impairment, and controls. The results showed that PAI-1 serum levels are increased in Alzheimer (23% more) and BDNF serum levels are decreased (13.7% less) as compared to amnestic mild cognitive impairment patients. In addition, the PAI-1/BDNF ratio was significantly increased in Alzheimer patients as compared to amnestic mild cognitive impairment (36.4% more) and controls (40% more). Lastly, the PAI-1/BDNF ratio negatively correlated with the Mini-Mental score.

### Elevated PAI-1 Levels and PAI-1/BDNF Ratio and Reduced BDNF Levels in Alzheimer’s Dementia

PAI-1 serum levels are increased in Alzheimer patients with dementia and amnestic mild cognitive impairment as compared to controls, a result in line with several previous observations in Alzheimer animal models and humans.^26-30^ As a consequence, tPA/plasmin synthesis in Alzheimer could be reduced, favoring the accumulation of β-amyloid and Alzheimer development.^24^

When we calculated the molar PAI-1/BDNF ratio, we found higher percentages of changes among groups. The PAI-1/BDNF ratio in Alzheimer patients was increased by 36.4% versus amnestic mild cognitive impairment patients and by 40% in controls. This finding suggests that measurement of this ratio may provide an indication of the degree of cognitive impairment in Alzheimer possibly caused by a concomitant PAI-1 increase and reduced conversion of proBDNF to mature BDNF.^3,36-39^ Supporting this hypothesis, BDNF serum levels were significantly lower in Alzheimer patients with dementia as compared to amnestic mild cognitive impairment patients (13.7% less), a finding consistent with previous studies reporting reduced levels of BDNF in Alzheimer patients.^21,40-42^

Furthermore, we also found that the PAI-1/BDNF ratio negatively correlated with the Mini-Mental score, which is a measure of cognitive function.

### Elevated PAI-1 Levels and/or Reduced BDNF Levels in Other Mental Illnesses

The consequences of increased PAI-1 expression, leading to decreased fibrinolytic/proteolytic activity, are relevant not only to Alzheimer’s disease but also to other mental disorders. Many studies have shown that inhibition of fibrinolysis/proteolysis can be associated with affective disorders such as depression^13,43^ and anxiety.^44^ Interestingly, decreased BDNF brain and circulating levels have been reported in depressive disorders.^45^ Accordingly, it has been hypothesized that increased PAI-1 levels, by inhibiting the conversion of proBDNF to mature BDNF, may contribute to brain dysfunctions observed in depression, including disrupted neurogenesis, synaptic plasticity, and reward processing.^46^ Further confirmation of the link between PAI-1 and depression comes from preclinical and human studies showing that antidepressants such as escitalopram produce downregulation of PAI-1 serum levels.^47^

## Implications of Our Findings

Our data on BDNF and PAI-1 serum do not provide per se new insights into the individual roles of these proteins in Alzheimer pathophysiology. However, our correlation analyses suggest a possible functional connection between these two proteins in Alzheimer. Understanding the molecular mechanisms underlying the regulation of BDNF by tPA/plasmin may provide new insights into the pathogenesis of Alzheimer’s disease and identify new potential therapeutic targets.

One possibility is to reduce PAI-1 levels with lifestyle and dietary interventions. Overweight and diabetes, in addition to being risk factors for Alzheimer’s disease, are conditions associated with high levels of PAI-1. In recent clinical trials, it was shown that a starch- and sucrose-reduced diet leads to decreased PAI-1 levels.^48^ Furthermore, in several studies, lifestyle- and dietary-mediated weight losses in overweight and moderately obese subjects have been associated with reductions in PAI-1 levels.^49^ Also, the use of anticoagulants can reduce PAI-1 levels^50^ and produce beneficial effects on Alzheimer, as recently hypothesized by Toribio-Fernandez and co-workers.^51^ In theory, the possibility to reduce PAI-1 levels in Alzheimer could have positive effects on cognitive functions, and these effects may involve an increase in mature BDNF due to increased tPA/plasmin activity.

Another possible implication of our findings is to use the PAI-1/BDNF ratio as a selective marker of Alzheimer dementia able to distinguish from other prodromal Alzheimer stages and/or cognitively healthy subjects. Interestingly, this increase in the PAI-1/BDNF ratio is selectively present in Alzheimer patients with full dementia and not in amnestic mild cognitive impairment and controls. The strategy of using the ratio between two proteins of the plasmin-BDNF pathway in serum has also been adopted in people affected by different mental disorders. The results demonstrated that the combination of multiple serum protein levels in this pathway was better than any single protein measurement in accuracy of diagnosis and differentiation of such disorders.^52,53^ Our results suggest that also in Alzheimer, the use of the ratio PAI-1/BDNF could be useful as a diagnostic marker of dementia when compared to single measurements of these two proteins. Nonetheless, other studies in larger cohorts of subjects are needed to confirm this assumption.

## Limitations of Our Study

There are relevant limitations to the interpretation of our data. The number of subjects included in the experimental groups is small. For this reason, our data should be considered preliminary. The main problem was the recruitment of mentally healthy subjects who wanted to undergo our routine biochemical, neurological, and neuropsychological tests. Thus, our data need to be confirmed in larger cohorts of subjects before we draw definitive conclusions. Furthermore, it should be noted that reduced tPA/plasmin activity in Alzheimer may also occur through mechanisms independent of inhibitory action of PAI-1. It has been shown that hyperhomocysteinemia can inhibit the binding of tPA with its receptor annexin II, thereby reducing plasmin synthesis.^54-56^ In line with these findings, in a recent meta-analysis and meta-regression of case-control studies, it has been reported that in Alzheimer patients, there is an approximate one-third increase in blood homocysteine, independently of disease severity.^57^

## Conclusions

In conclusion, this study shows that BDNF and PAI-1 are dysregulated in an opposite direction in Alzheimer. More studies are needed to provide new insights into the relationship among BDNF, PAI-1, and their ratio in Alzheimer.

## List of abbreviations

ANOVA: univariate analyses of variance
BDNF: brain-derived neurotrophic factor
CNS: central nervous system
MRI: magnetic resonance imaging
PAI-1: plasminogen activator inhibitor-1
tPA: tissue plasminogen activator
